# Reducing maternal mortality in Sub-Saharan Africa through emergency obstetric care

**DOI:** 10.18332/ejm/140130

**Published:** 2021-09-08

**Authors:** Rafiat T. Akinokun

**Affiliations:** 1 Ladoke Akintola University of Technology, Ogbomoso, Nigeria

**Keywords:** Africa, emergency obstetric care, maternal mortality

**Dear Editor,**

The highest rates of maternal and infant mortality have been recorded continuously in Sub-Saharan Africa because pregnant women in this region have difficulty receiving expert emergency obstetric care due to the delay in seeking appropriate medical help, delay in getting to an appropriate health facility, and delay in receiving expert medical help after reaching the appropriate health facility^1,2^. Sadly, efforts towards reducing maternal mortality in Sub-Saharan Africa have been inadequate. Pregnant women go through birthing at home and in mission houses because of poverty and poor transportation facilities to healthcare centers. In addition, poor staffing and lack of essential drugs and equipment in the healthcare centers contribute to poor obstetric healthcare delivery^3^.

Pregnant women should have access to emergency obstetric care and should be attended by skilled health personnel, either a doctor, nurse, or midwife^4^. According to the World Health Organization, Five Emergency Obstetric care facilities including at least one comprehensive facility should serve a population of about 0.5 million people^5^. Meeting the obstetric care needs of pregnant and postpartum women should start from the foundation. Pregnant women in communities should be assisted by the midwife to develop a birth preparedness and complications readiness plan with the knowledge that every pregnant woman is at risk of developing complications. A key aspect of this is making pregnant women aware of danger signs in pregnancy while having a transportation plan in case they experience any of the danger signs.

Nurses and midwives operating at every level of the healthcare delivery system should always be prepared to attend to emergency obstetric cases. The SHARP principle is a recommended format for midwives providing basic emergency obstetric care, which is expanded as:

1. **S**eek help when attending to emergencies;
2. **H**ave what you need, including the pregnancy history, test results, sutures for episiorrhaphy, and sterile instruments for assisted delivery;
3. **A**ctivate emergency responses such as administration of uterotonics, antibiotics, and anticonvulsants for required cases;
4. **R**efer cases requiring comprehensive emergency obstetric care such as Caesarian section and blood transfusion to the nearest comprehensive healthcare center;
5. **P**repare the pregnant woman for surgery, specifically for nurses and midwives in centers providing comprehensive emergency obstetric care.

Meeting the emergency needs of pregnant women is a collaborative effort of the community, the skilled healthcare provider, and health systems. Investments should be made to ensure that primary healthcare centers are continuously equipped with essential resources to meet the ever-growing demand for obstetric care in Sub-Saharan Africa. Maternal mortality is painful and no woman should die in the process of bringing another human to life. While efforts are continuously made to reduce maternal mortality in the Sub-Saharan African region, midwives should continue to strive to meet the emergency needs of pregnant and postpartum women to reduce maternal mortality.

## Data Availability

Data sharing is not applicable to this article as no new data were created.

## References

[1] (2019). Maternal mortality.

[2] The Three Delays Model and our Integrated Approach.

[3] Mkoka DA, Goicolea I, Kiwara A, Mwangu M, Hurtig AK (2014). Availability of drugs and medical supplies for emergency obstetric care: experience of health facility managers in a rural District of Tanzania. BMC Pregnancy Childbirth.

[4] World Health Organization (2004). Making pregnancy safer: the critical role of the skilled attendant: A joint statement by WHO, ICM and FIGO.

[5] World Health Organization (2009). Monitoring emergency obstetric care: a handbook.

